# Supplementary material for: Negotiating Complexity: Challenges to Implementing Community-Led Nature-Based Solutions in England Pre- and Post-COVID-19
Source: Int J Environ Res Public Health. 2022 Nov 12;19(22):14906. doi: 10.3390/ijerph192214906 (PMC9691162; doi:10.3390/ijerph192214906)
Supplement: Supplementary file 1 [file ijerph-19-14906-s001.zip › Table S1_Interview Questionnaire.pdf]

Interview Questionnaire

General Questions: For all participants. These are intended to be followed if necessary by more targeted ones according to the field of expertise or current role of the participant

Role and relationship with Mental Health / Wellbeing / NbS

1. What is your field of expertise, role and responsibilities within [organisation]?
2. Can you briefly describe the role / purpose of your organisation / practice?
3. How, if at all, has the focus / aims of your organisation shifted as a result of COVID-19?
4. Based on what you just said, would you consider your organisation /practice's work to be related to mental health and wellbeing?
5. If so, what kind of work are you doing to promote mental health and wellbeing within your organisation/ practice?

Organisation / Practice and COVID-19- Challenges and Barriers

6. What would you say are the challenges and barriers facing the local communities for COVID-19 recovery in terms of public health and wellbeing? (In reference to the general population not the disease itself)
7. What would you say are the challenges and barriers facing your organisation / practice aims?
8. How, if at all, would you say those challenges and barriers have changed due to COVID-19?
9. What needs to happen for these barriers and challenges to be overcome?
10. Has your approach to mental health and wellbeing changed due to COVID-19?
11. Would you said there have been a change in approach from local authorities / social care organisation / charities / other stakeholders towards wellbeing and mental health due to COVID-19

Organisation / Practice and NbS

12. Has your organisation's involvement with NbS changed (increased/decreased – started/ended) due to COVID-19?
13. Is any of this work purposely done to address mental health and wellbeing issues?
14. Is there anything preventing you from actively incorporating NbS into your organisation/practice?
15. What would you say are the challenges and barriers to integrating NbS into your organisation/ practice?
16. How would you say those challenges and barriers have changed due to COVID-19?
17. What needs to happen for these barriers and challenges to be overcome?
18. How, if at all, would you say NbS offer new / alternative ways of meeting COVID-19 recovery challenges?
19. How, if at all, do you think COVID-19 has impacted NbS development and implementation?

Targeted Questions

Healthcare Practitioners

20. How, if at all, would you say NbS relates to healthcare?
21. Are you aware of the Social prescribing programmes being offered by local authorities or the NHS?
22. If so, how do you understand Social prescribing? What about Green social prescribing?

23. Is there anything preventing you from actively incorporating green social prescribing as part of your regular practice?
24. What benefits, if any, can you see in incorporating green social prescribing in your practice?
25. Are there any conditions/situations for which you would choose to provide a green social prescription?
26. Are you aware of any NHS guidelines that recommend green social prescription? If so, are these prescriptions being recommended as a treatment for any particular ailments?
27. What kind of work, if any, are you doing to promote integration of NbS within health practice?
28. Is there anything preventing you from actively incorporating green social prescribing into your practice?
29. What would you say are the challenges and barriers of integrating green social prescribing into health practice?
30. Would you say those challenges and barriers have changed due to COVID-19?
31. What needs to happen for these barriers and challenges to be overcome?

Social Prescribing Practitioners / Advocates

32. From your own experience, how does social prescribing work? (We want to understand who uses it as part of their practices and who is it aimed at)
33. What are the main issues currently being addressed with social prescribing?
34. How, if at all, has your incorporation / usage of social prescribing changed due to COVID-19?
35. Since COVID-19, are there any additional / new benefits you could identify for using social prescribing?
36. How, if at all, would you say NbS relates to social prescribing?
37. What would you say are the challenges and barriers to using social prescribing regularly?
38. How would you say those challenges and barriers have changed due to COVID-19?
39. What needs to happen for these barriers and challenges to be overcome?

Funding Body

40. What is unique about the purpose of your organisation?
41. What are your organisation's current funding priorities?
42. How, if at all, has this changed since the onset of COVID-19?
43. Do you have any population groups / types of organisation targeted as suitable applicants for funding?
44. What main criteria do applicants need to reach in order to qualify i.e. what core elements are you looking for?
45. What key objectives are applicants aiming to achieve?
46. Have you been funding projects aiming to deliver / implement NbS for health and wellbeing purposes? If yes, why? Do these projects make up a significant proportion of your overall funded projects?
47. What do you think these projects bring to the communities that access them?
48. What is your hope for these projects?
49. Has your organisation promoted specific funding opportunities aiming to address consequences of COVID-19?
50. How, if at all, has COVID-19 affected your funding distribution / schemes? I.e. the types of projects you support / types of projects requesting funding.

Government

Local Government

51. Which would you say are the most pressing needs facing your local communities? Have these changed due to COVID-19?
52. What kind of NbS, if any, are being implemented in your area for addressing public health and wellbeing issues?
53. Has your organisation had any involvement in local or regional NbS developments? (advocate, visit, collaborate directly, take part, volunteer)
54. What NbS solutions, if any, would you like to see implemented in your local area for addressing public health and wellbeing issues?
55. What benefits, if any, can you see in developing/supporting NbS in your local area?
56. How, if at all, are NbS approaches being incorporated into local policy?
57. In your opinion, who do you think, if anyone, should take the lead in developing NbS in your area / within local communities?
58. What, if any, should be the role of the local government in developing, implementing and maintaining NbS?
59. Do you think community based organisations have the opportunity to lead the way in making NbS mainstream?
60. What would you say are the challenges and barriers to developing / implementing / incorporating NbS approaches to your local area / policies?
61. How would you say those challenges and barriers have changed due to COVID-19?
62. What needs to happen for these barriers and challenges to be overcome?

Central Government / Covid Recovery Board

63. What strategies / policies are in place for tackling those challenges?
64. To what extent, if at all, are NbS considered as part of those strategies / policies?
65. What kind of NbS approaches, if any, are being implemented regionally or nationally, that you are aware of?
66. How, if at all, are NbS approaches being incorporated into regional / national policy?
67. In your opinion, who do you think, if anyone, should take the lead in developing NbS at a regional / national level?
68. Do community based organisations have a role in developing / implementing / disseminating NbS solutions at a regional / national level?
69. What would you say are the challenges and barriers to developing / implementing / incorporating NbS approaches into regional or national / policies?
70. How would you say those challenges and barriers have changed due to COVID-19?
71. What needs to happen for these barriers and challenges to be overcome?

Closing questions for all:

72. What single change would you say could significantly improve or increase the use of NbS / Social prescribing for public health and wellbeing purposes?
73. What is your key message about NbS in relation to health and wellbeing?
74. Finally, as part of the outcomes of the project we are developing a platform that is so far in the research phase. (this includes for e.g. mapping, knowledge share)/ What kind of platform do you think would be useful to support connecting people with nature for health and wellbeing?
75. Will you be happy to be kept in the loop regarding the outcomes of the project?
76. Would you be happy to be contacted again?
